# Unique evolutionary trajectories of breast cancers with distinct genomic and spatial heterogeneity

**DOI:** 10.1038/s41598-021-90170-1

**Published:** 2021-05-19

**Authors:** Tanya N. Phung, Timothy H. Webster, Elizabeth Lenkiewicz, Smriti Malasi, Mariacarla Andreozzi, Ann E. McCullough, Karen S. Anderson, Barbara A. Pockaj, Melissa A. Wilson, Michael T. Barrett

**Affiliations:** 1 School of Life Sciences, Arizona State University, Tempe, AZ USA; 2 Center for Evolution and Medicine, Arizona State University, Tempe, AZ USA; 3 Department of Anthropology, University of Utah, Salt Lake City, UT USA; 4 Division of Hematology/Oncology, Department of Internal Medicine, Mayo Clinic, Scottsdale, AZ USA; 5 Department of Pathology and Laboratory Medicine, Mayo Clinic in Arizona, Scottsdale, AZ USA; 6 Biodesign Institute, Arizona State University, Tempe, AZ USA; 7 Division of General Surgery, Section of Surgical Oncology, Mayo Clinic in Arizona, Phoenix, AZ USA

**Keywords:** Cancer, Computational biology and bioinformatics, Oncology

## Abstract

Breast cancers exhibit intratumoral heterogeneity associated with disease progression and therapeutic resistance. To define the sources and the extent of heterogeneity, we performed an in-depth analysis of the genomic architecture of three chemoradiation-naïve breast cancers with well-defined clinical features including variable ER, PR, ERBB2 receptor expression and two distinct pathogenic BRCA2^mut^ genotypes. The latter included a germ line carrier and a patient with a somatic variant. In each case we combined DNA content-based flow cytometry with whole exome sequencing and genome wide copy number variant (CNV) analysis of distinct populations sorted from multiple (4–18) mapped biopsies within the tumors and involved lymph nodes. Interrogating flow-sorted tumor populations from each biopsy provided an objective method to distinguish fixed and variable genomic lesions in each tumor. Notably we show that tumors exploit CNVs to fix mutations and deletions in distinct populations throughout each tumor. The identification of fixed genomic lesions that are shared or unique within each tumor, has broad implications for the study of tumor heterogeneity including the presence of tumor markers and therapeutic targets, and of candidate neoepitopes in breast and other solid tumors that can advance more effective treatment and clinical management of patients with disease.

## Introduction

It is well established that tumors arise as a result of an acquired genomic instability and the evolution of distinct cell lineages during the natural history of disease^1–3^. Consequently, tumors have unique patterns of mutations and copy number aberrations in their genomes. The nature and the extent of tumor heterogeneity within a tumor are challenges for advancing improved more personalized care for patients with disease. Next generation sequencing (NGS) surveys of one or more regions within tumors have described a complex genomic landscape for breast and other solid tumors^1,4,5^. Notably, NGS studies have used variant allele fractions (VAFs) to reconstruct the clonal composition of tumors of interest^6,7^. A recurrent theme in these studies is that tumors consist of cell lineages with tree-like structures composed of clonal (trunk) and sub clonal (branches) mutations. A fundamental hypothesis is that the composition and heterogeneity of the tumor cell lineages present in a patient’s tumor will impact its’ clinical history^8–10^. Thus, it has been proposed that measures of intra tumor heterogeneity based on multiple biopsies are needed to accurately stage a tumor and to identify those genomic lesions that can be exploited for improved patient care^5,9^.

Clinical management of breast cancer patients relies on the status of estrogen (ER), progesterone (PR), and ERBB2 receptors in diagnostic biopsies. Results for these markers are typically based on well-established IHC and FISH assays that incorporate staining intensity and the percentage of positive cell numbers for each sample of interest^11,12^. Heterogeneous results for one or more of these clinical markers are often observed in different sites within a tumor, suggesting that multiple clonal populations with unique and shared driver mutations and CNVs may be present. In addition to receptor status pathogenic BRCA1/2 mutations associated with homologous recombination deficiency (HRD) define a distinct clinical class of breast tumors^13,14^. The latter are characterized by elevated levels of genomic instability, a hallmark of cancer, notably an increase in intra chromosomal CNV aberrations relative to BRCA^wt^ tumors^15^. Thus, BRCA2^mut^ tumors that express an HRD phenotype represent an additional challenge for assessing tumor heterogeneity and accurate clinical staging.

The loss of normal cell cycle control and the development of tetraploid and aneuploid cell populations arise in premalignant tissues and contribute to the evolution of neoplasias^3,16,17^. In addition to VAFs of single nucleotide variants (SNVs), tumors display highly variable patterns of CNVs including both gains and losses. These can range from single copy changes spanning whole chromosomes to focal high-level amplifications and intragenic homozygous deletions. The presence of CNVs alters the ratio of alleles, notably in non-diploid tumors, and may provide highly selectable changes in the dosages of oncogenes and tumor suppressors throughout the clinical history of a tumor. Current models of tumor heterogeneity rely on bulk tumor preparations, pathological review, and the application of multiple computational tools to deconvolute the presence of clonal populations^5,18,19^. Tumor lineages are described based on predicted shared clonal events and include genes with lesions described as double hits (e.g., variant plus copy number loss, biallelic variant, homozygous deletion) in histologically admixed samples. However genomic instability including ploidy and chromosome instability, and variable admixtures of non-neoplastic cells in samples of interest limit the precision and potential clinical impact of VAF based methods in the identification of shared genomic lesions in heterogeneous tumors.

Fixed variants, notably homozygous SNVs and deletions, are those that are present in the absence of an alternative variant and thus cannot revert or be replaced by a preexisting allele. These variants can dramatically affect the natural or clinical history of a tumor. Of significant interest are those fixed variants targeting clinically relevant genes that are shared across the populations or that distinguish unique populations within a tumor. The emergence and persistence of fixed variants in aneuploid genomes can be driven by changes in ploidy and both the nature and extent of genomic instability that drives the tumor. Thus, in contrast to variants mapping to autosomes and sex chromosomes in normal diploid cells, the identification of fixed variants in tumor genomes needs to account for the presence of abnormal genotypes, chromosomes, and ploidies present in histologically admixed tumors.

In this study we systematically interrogated the composition of 3 chemoradiation naïve surgically excised ductal carcinomas with well annotated clinical and genomic features. To overcome the limitations of VAF based methods with bulk tumor samples we combined flow cytometry, whole genome CNV, and whole exome SNV analyses to identify fixed lesions within distinct aneuploid populations present in multiple (4–18) biopsies from each of the three heterogeneous breast tumors. The first tumor, PS13-9062, was a grade 3 ER+, PR+, and ERBB2- (pT2, pN0) arising in a patient with a germ line BRCA2 mutation. The second tumor, PS13-1760, was classified as a grade 3 node negative ER+ PR− ERBB2+ (3+) (pT2, pN0) and had a somatic BRCA2 mutation. The third tumor, PS13-585, was a large node positive tumor (pT2, pN3c, pMX) with a diagnosis based on a single core needle biopsy of grade 3 ER+ PR− ERBB2+ (3+). However, further IHC testing of mapped research biopsies scored ERBB2 as equivocal with heterogeneous staining within primary and lymph nodes. In addition, both ER and PR IHC staining were heterogeneous throughout the tumor with abrupt staining boundaries for both clinical markers. These three tumors with distinct clinical and genomic contexts provide a model to systematically study the nature and extent of heterogeneity based on mapped clonal aneuploid populations, shared and unique fixed somatic lesions, established clinical markers used for the management of patients with breast cancer, and the presence of predicted neoepitopes.

## Results

### Aneuploid populations within resectable ER+ breast tumors

We screened the mapped biopsies from each tumor with DNA content flow cytometry and sorted each diploid, tetraploid, and aneuploid population detected. Patient 1 (PS13-9062) had a single ploidy (3.7 N) in each of the 4 biopsies mapped within the tumor (Supplemental Fig. 1). The second patient (PS13-1750) had a dominant ploidy (3.2 N) in each of the 10 (A1–A10) primary tumor biopsies and a second co-existing ploidy (3.6 N) in one (A2) biopsy (Supplemental Fig. 2). Patient 3 (PS13-585) had 12 mapped biopsies (A1–A12) within the primary tissue, four (B1–B3, D1) from two adjacent nodes, and two (F1, F2) from a distal node. In total we detected six different non-diploid populations across the 18 mapped biopsies (Supplemental Fig. 3). Co-existing ploidies were present in six of the primary biopsies. In contrast the nodes contained either a 5.8 N (2/6 biopsies) or a 5.0 N (4/6 biopsies) population. Strikingly both populations were present as co-existing ploidies within biopsies of 2 regions (A3 and A4) in the primary. The presence of aneuploid populations with distinct ploidies provides an initial measure of heterogeneity in each tumor. In addition, these sorted populations provide templates for high-resolution analysis for the detection of fixed variants, SNVs and CNVs, and neoepitopes that are either shared or unique to each of the populations within each tumor.

#### PS13-9062

**Figure 1:**
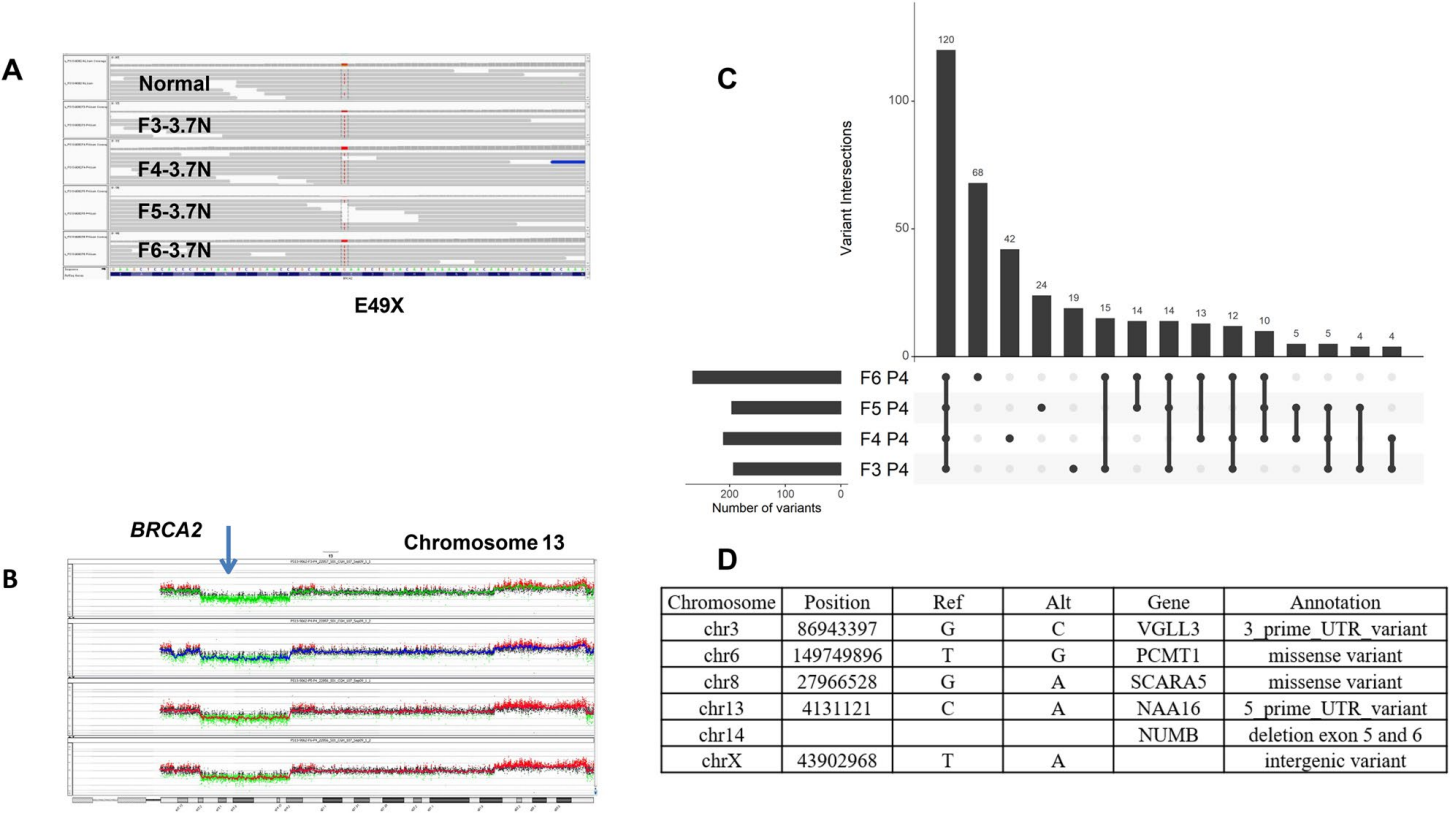
Genomic analysis of BRCA2 mutant germ line carrier PS13-9062. (**A**) IGV view of BRCA2 in normal and 3.7 N populations flow sorted from biopsies F3–F6. (**B**) Chromosome 13 CNV plots for biopsies F3–F6. Blue arrow denotes BRCA2 locus that maps to interstitial deletion of 13q12.3–q14.2 in each 3.7 N population. (**C**) Upset plot displaying patterns of variant sharing among samples in flow sorted biopsies. (**D**) Shared fixed somatic variants within the tumor.

This tumor arose in a patient with a known pathogenic *BRCA2*^E49X^ germ line variant. The genomes of each sorted population displayed multiple unique and shared CNVs that reflect the BRCA2 mutated status (Supplemental Fig. 4). Notably the *BRCA2*^E49X^ variant was fixed by an interstitial 13q12.3–q14.2 deletion in the 3.7 N tumor population present in each of the four biopsies (Fig. 1A, B). We detected a total of 369 somatic mutations across the 4 biopsies (Fig. 1C, D). Of these 120 (33%) were shared, while mutations unique to a single region varied from 19 in F3 (5.1%) to 68 in F6 (18.4%). Shared mutations that were fixed in each population included 3 variants in non-coding regions and missense mutations in a protein carboxyl methyltransferase, *PCMT1*, and the ferritin receptor *SCARA5*. In addition, there was a fixed *PIAS4*^N156S^ missense mutation present in the 4 biopsies, however one sample (F5) had coverage that was below our minimum NGS threshold of 10 reads. The latter gene, Protein Inhibitor of Activated STAT 4, is a SUMO E3-ligase that promotes responses to DNA double strand breaks^20^. In addition, we identified a shared fixed 15 kb deletion targeting exons 5 and 6 of *NUMB* in both our CNV and exome results (Supplemental Fig. 5). Decreased expression of Numb has been linked to activation of the proto-oncogene Notch1 and reduction in TP53^21^. Notably loss of Numb expression is a marker of tumor aggressiveness, potentially linked to BRCA status and a cancer stem cell phenotype in primary breast cancer. The fixed nature of the *PIAS4* and *NUMB* lesions supports a model suggesting that these likely promoted the BRCA2 phenotype of this tumor.

#### PS13-1750

**Figure 2:**
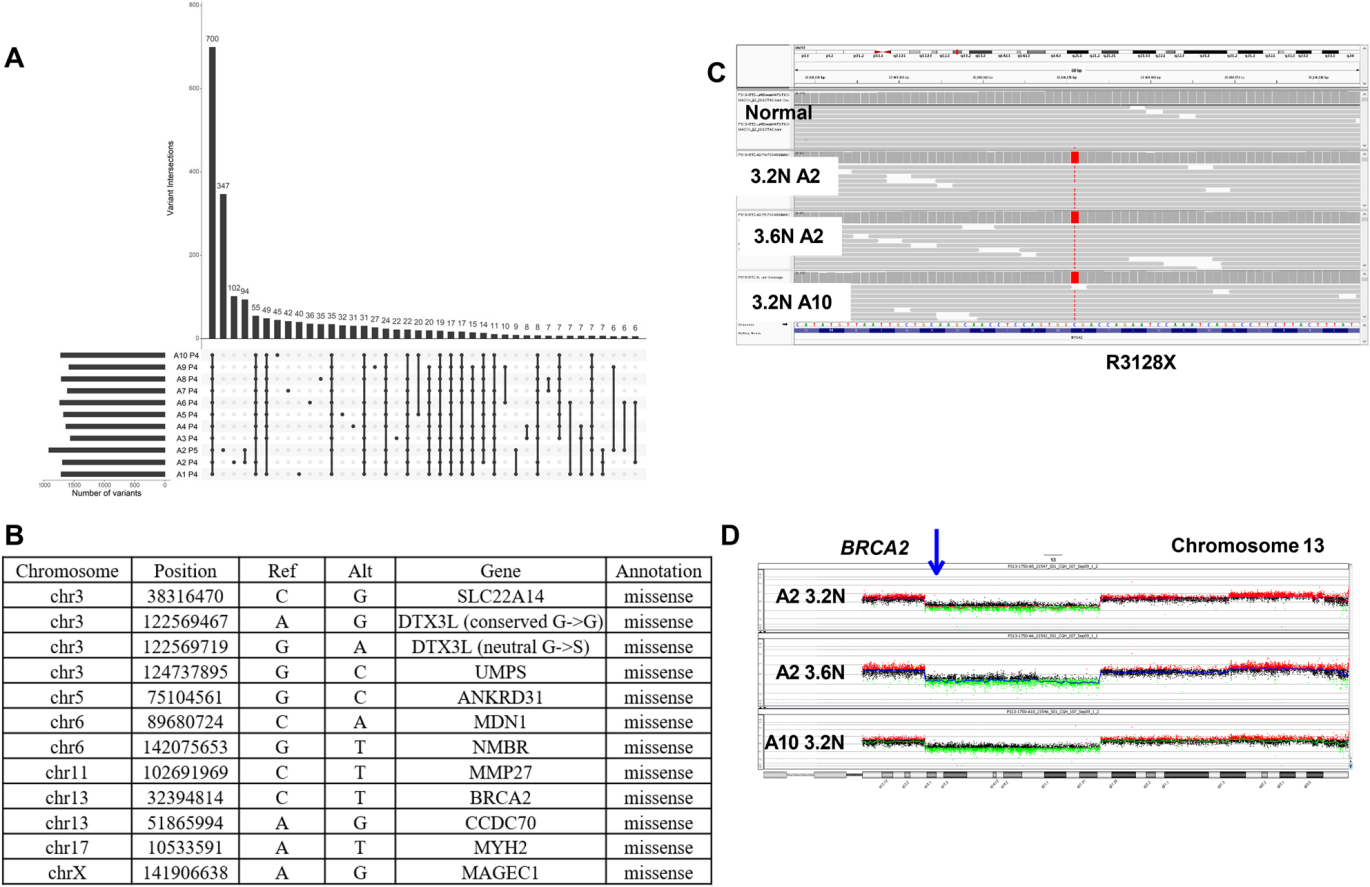
Genomic analysis of somatic BRCA2 mutant PS13-1750. (**A**) Upset plot displaying patterns of variant sharing among samples in flow sorted biopsies. (**B**) Shared fixed coding and non-synonymous somatic variants within the tumor. (**C**) IGV view of BRCA2 in normal, 3.2 N populations flow sorted from biopsies A2 and A10, and 3.6 N population in A2. (**D**) Chromosome 13 CNV plots for 3.2 N populations flow sorted from biopsies A2 and A10, and 3.6 N population in A2. Blue arrow denotes BRCA2 locus that maps to interstitial deletion of 13q13.1–q21.32 in each population.

The tumor has 3105 somatic mutations including 700 (23%) that were shared across the ten biopsies and the eleven sorted populations (Fig. 2A). Of these 84/700 (12%) were fixed. The majority 59/84 (70%) of these targeted non-coding regions while twenty-five targeted coding sequences. The latter included thirteen synonymous variants and twelve non-synonymous variants. One of the latter was the pathogenic nonsense variant *BRCA2*^R3128X^, while eleven were missense variants of which only two, *UMPS*^G213A^ and *NMBR*^L390M^, had predicted pathogenicity (Fig. 2B). The shared variants mapped primarily to regions of deletions such as 13q13.1–q21.32 that included the BRCA2 locus (Fig. 2C, D). The CNV patterns were analogous to, but distinct from, those in the BRCA2 germ line carrier PS13-9062 with variable shared and unique CNVs across the sorted populations in each tumor (Supplemental Fig. 6).

**Figure 3:**
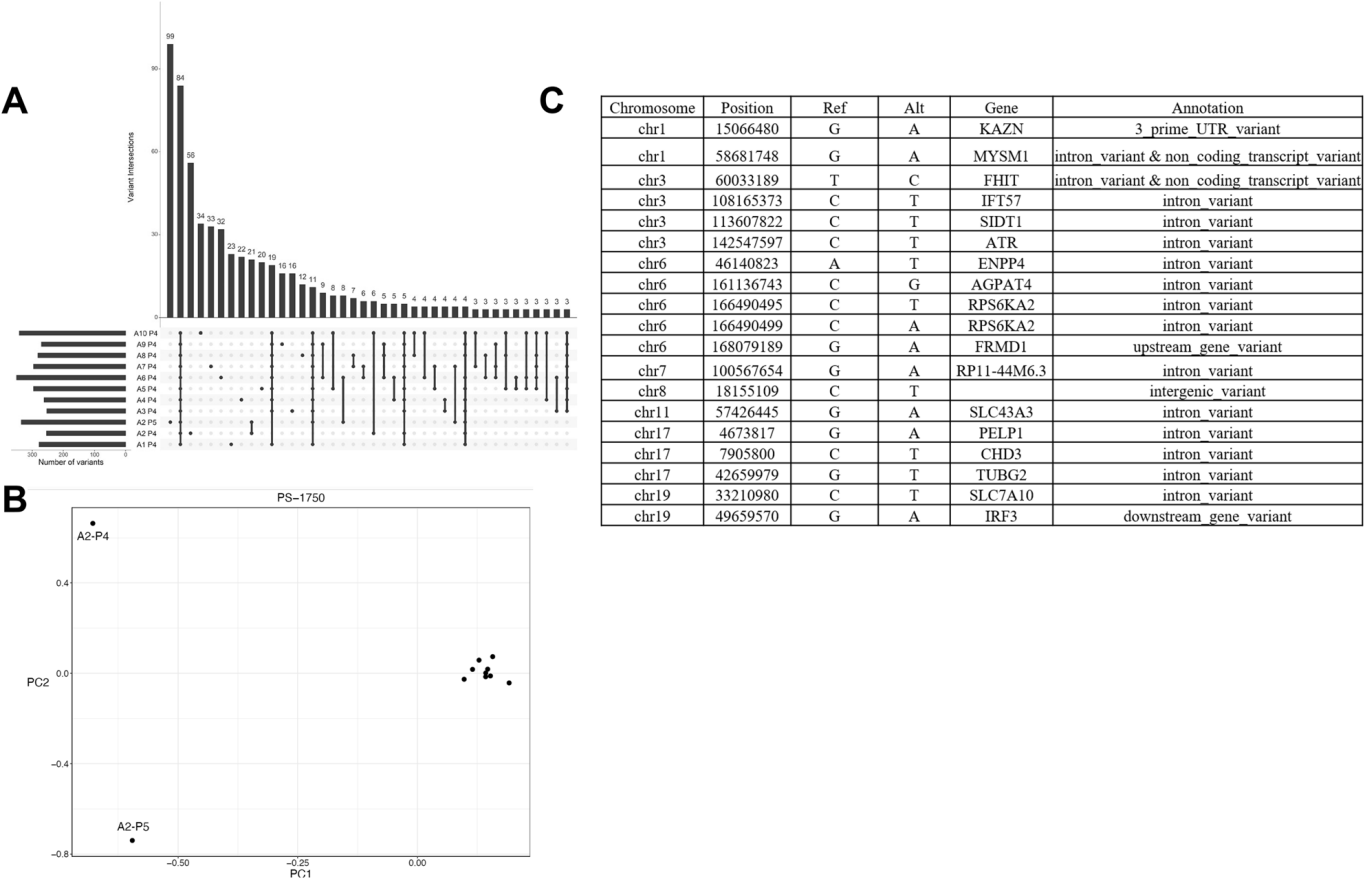
Shared and unique fixed variants in PS13-1750. (**A**) Upset plot displaying patterns of variant sharing among samples in flow sorted biopsies. (**B**) Principal component analysis (PCA) distinguishes co-occurring 3.2 N and 3.6 N populations in biopsy A2. (**C**) Unique fixed variants in 3.6 N population.

Strikingly, the co-existing ploidies A2-3.6 N and A2-3.2 N contained the most unique variants, 347 and 102 respectively (Fig. 2A). In addition, the unique ploidy A2-3.6 N had the highest number (99) of unique fixed variants (Fig. 3A). Principle component analysis (PCA) with the somatic variants distinguished these two ploidies in A2 from each other and from the other 9 populations in the tumor (Fig. 3B). Many of these fixed mutations were present as heterozygous variants in the other populations and were fixed because of somatic CNVs, the majority of which were losses. However, 19/99 of the mutations that were uniquely fixed in A2-3.6 N were absent in the other nine populations. These 19 de novo variants were present in non-coding regions including those of tumor associated genes *ATR*, *RPS6KA2*, *IRF3*, and *CHD3* (Fig. 3C)^22–26^.

#### PS13-585

**Figure 4:**
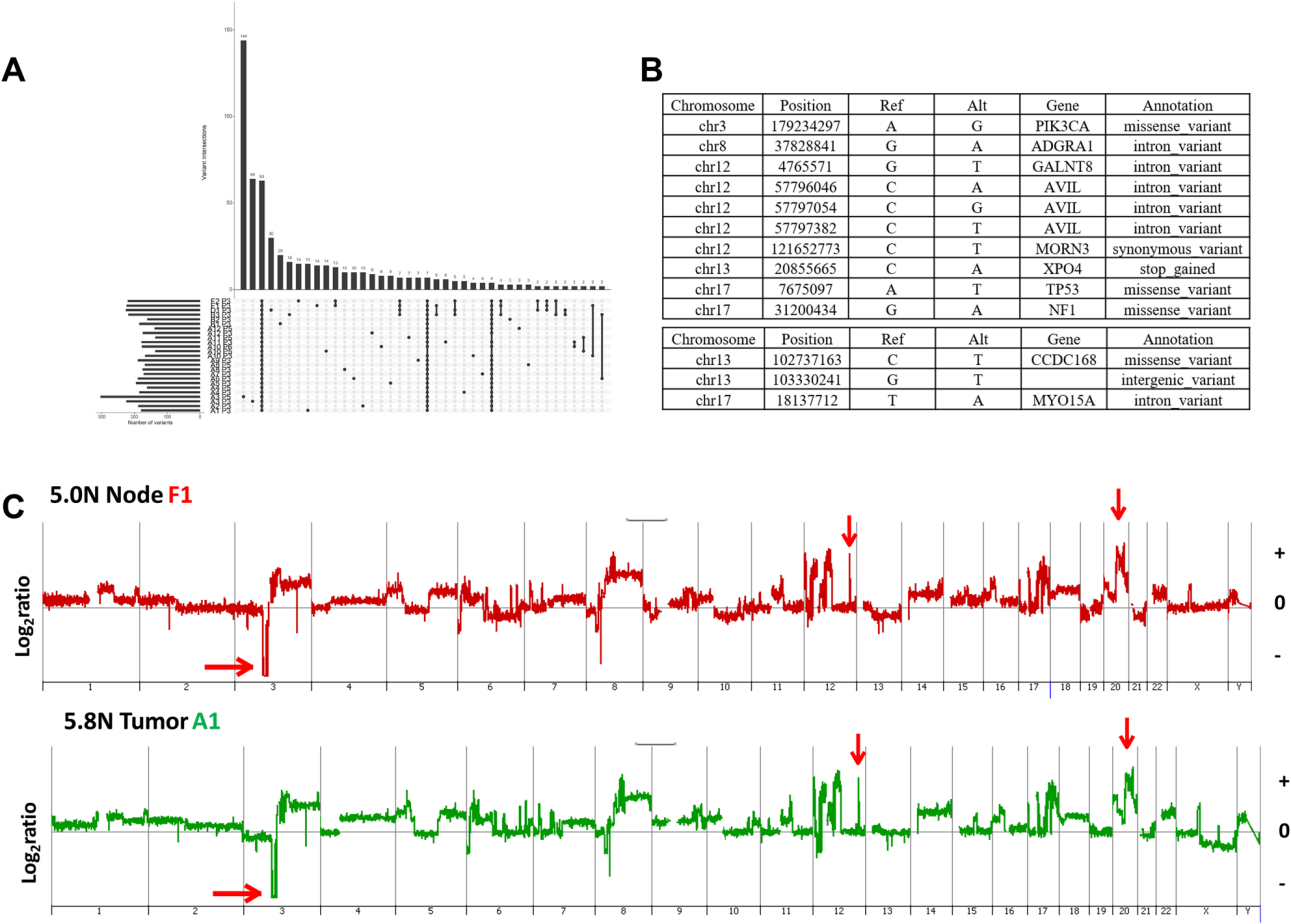
Shared and unique fixed variants in PS13-585. (**A**) Upset plot displaying patterns of variant sharing among samples. (**B**) Shared fixed somatic variants within the tumor (top) and node-specific shared fixed variants (bottom). (**C**) CNV plots of 5.8 N population from primary biopsy A1 and of 5.0 N population from distant node F1. Arrows denote shared focal CNVs homozygous deletion of 3p, and focal amplicons at 12q and 22q.

We detected a total of 801 somatic mutations across the 25 populations sorted from the 18 biopsies. Of these only 63 (8%) were shared, while mutations unique to a single region varied extensively across the tumor notably within the primary tumor where the co-existing 5.0 N and 5.8 N ploidies in A3 had 144 and 64 unique variants respectively (Fig. 4A). In contrast the same ploidies in the adjacent A4 region had only 5 or no unique variants. Ten fixed variants were shared within the primary tumor and the nodes notably pathogenic missense variants in *TP53*^V172D^, *PIK3CA*^H1047R^, and *NF1*^D301N^ (Fig. 4B). Targeted resequencing of these three genes in single nuclei sorted from 5.0 N populations within the primary and lymph nodes confirmed the fixed nature of the three pathogenic variants in single cells (Supplemental Fig. 7). In contrast, the presence of a non-fixed *APC*^D953V^ variant included fixed, heterogeneous, and wild type single nuclei in the primary and node samples. In addition a novel nonsense mutation in *XPO4*^E140X^ (Exportin 4) a member of a family of nuclear transporters, known to export Smad3 (a component of TGFβ signaling) and Eif5a1 and Eif5a2 (two closely related translation initiation factors) from the nucleus, was also shared^27,28^. This gene has been identified as a tumor suppressor in hepatocellular carcinoma^29^. Four additional shared fixed variants within non-coding regions mapped to the two highest amplicons across the tumor on chromosome 12p13.32 and 12q14.1 and reflect the stability of these CNVs. There were 3 additional node-specific shared fixed variants including two on chromosome 13, a missense variant *CCDC168*^D4512N^ and a variant in an intergenic region, and an intronic variant of *MYO15A* at 17p11.2.

A striking finding in the BRCA^WT^ tumor PS13-585 was the presence of aberrant CNV patterns but stable genomes across the primary and nodal tissues and multiple ploidies (Fig. 4C). The genomes of each of the 24 sorted aneuploid populations in this tumor shared a high level 20q11.23–q12 amplicon targeting *SRC*, a chromosome 12 with multiple amplicons and deletions, including a large homozygous deletion spanning 9.5 Mb at 3p12.1–p12.3 that targeted the axon guidance Roundabout Guidance Receptors 1 and 2 (*ROBO1* and *ROBO2*) loci (Supplemental Figs. 8, 9). In each case the boundaries of the aberrant intervals, including amplicons of variable heights and lengths, partial and homozygous deletions were conserved across the different ploidies and tissues.

### Clinical markers

**Figure 5:**
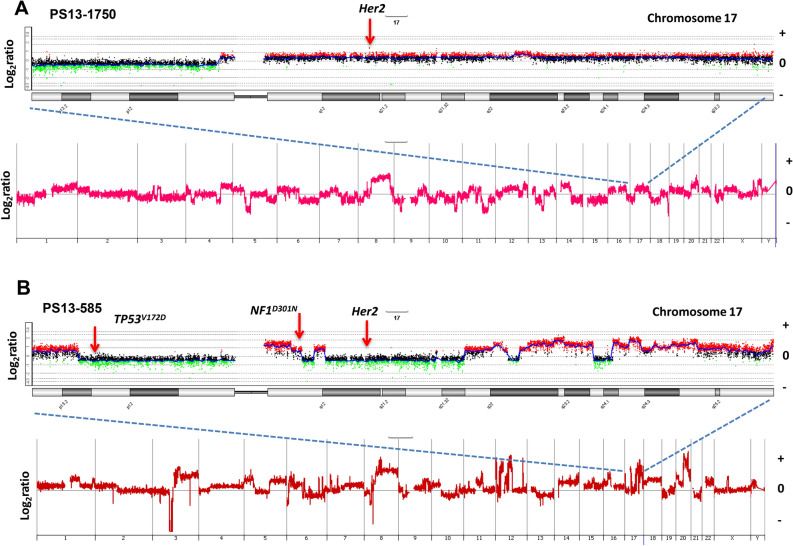
Genomic analysis of ERBB2 in PS13-1750 and PS13-585. Whole genome and chromosome 17 CNV plots of (**A**) PS13-1750 and (**B**) PS13-585. Both tumors were scored as ERBB2 3+ by IHC of single biopsies.

Two of the three tumors, PS13-1750 and PS13-585, were scored as ER+ PR– ERBB2+ (3+) based on clinical evaluation of a single biopsy. However, further IHC testing of mapped research biopsies from P13-585 scored ERBB2 as equivocal with heterogeneous staining within primary and lymph nodes. We did not detect evidence for ERBB2 amplification in any of the sorted samples from both cases (Fig. 5). In PS13-1750 there was a uniform low-level copy number gain spanning 17p11-qtel in each sorted population (Fig. 5A). In comparison chromosome 17 had multiple CNVs on both arms in each of the sorted populations and multiple ploidies present in PS13-585 (Fig. 5B). However, chromosome 17, which contains two of the shared fixed pathogenic somatic variants, *TP53*^V172D^ on the p arm and *NF1*^D301N^ on the q arm, was stable with a gain of 17q11.2 relative to 17q12–q21.32. Thus, in both tumors there was no evidence for selective ERBB2 amplicons in any of the flow sorted samples.

### Neoepitope predictions

**Figure 6:**
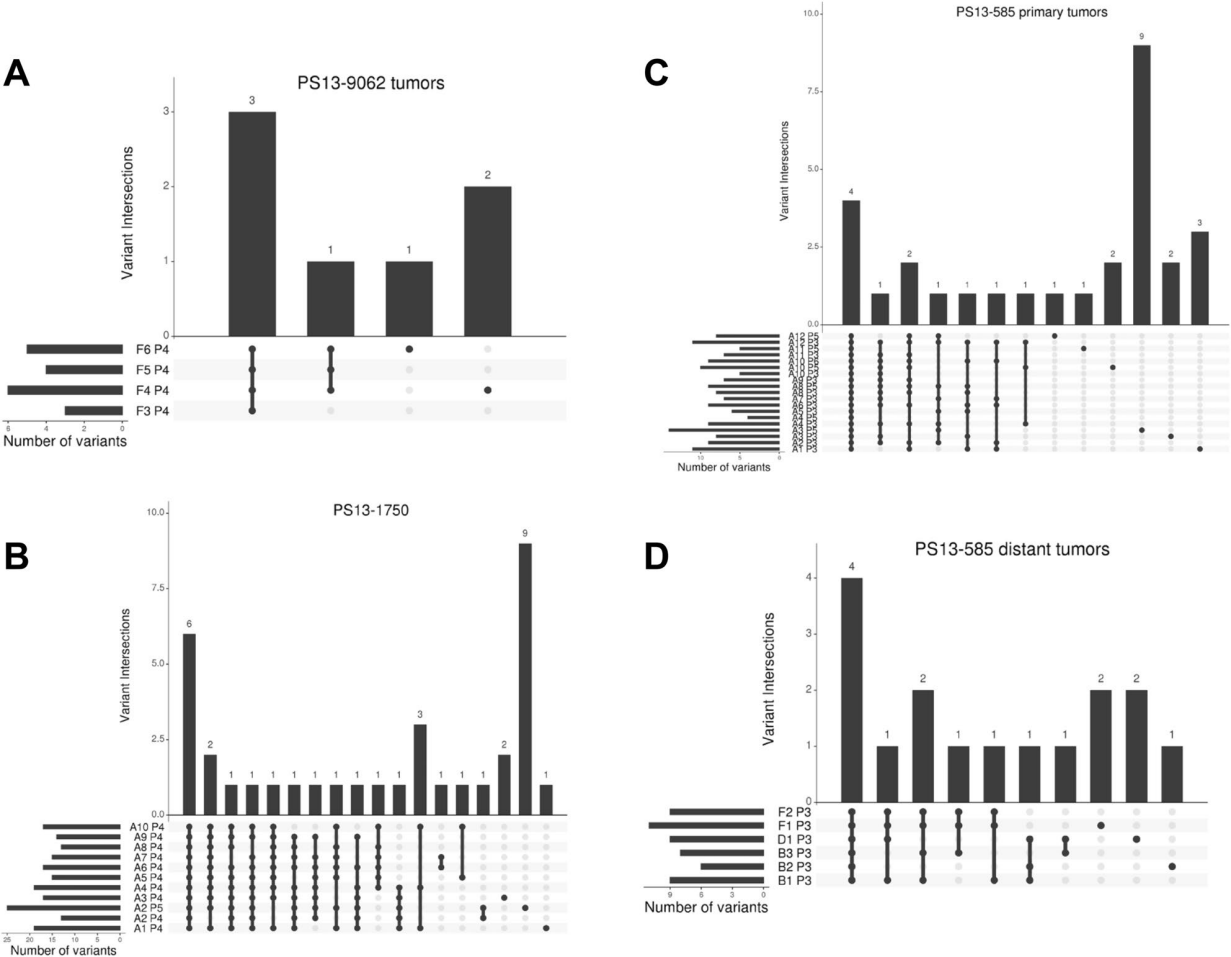
Predicted neoepitopes in each tumor. Upset plots of shared and unique predicted epitopes in (**A**) PS13-9062, (**B**) PS13-1750, and PS-585, (**C**) primary and (**D**) lymph nodes.

We applied our established neoepitope prediction pipeline with the total missense mutations in each of the three tumors as inputs^30^. Multiple variants were identified in the three tumors with each sorted population having one or more predicted neoepitope (Fig. 6; Table 1). PS13-9062, a pathogenic BRCA2 germ line carrier, had three shared predicted neoepitopes that included Prostaglandin F Receptor (*PTGFR*). The 10 biopsies and 11 sorted aneuploid populations in PS13-1750, containing a somatic pathogenic BRCA2 variant, had six shared predicted neoepitopes derived from variants in genes with diverse functions including a transcription factor (*PHTF2*), a regulator of apoptosis (*DAP*), and a protein tyrosine phosphatase (*MTMR11*). Strikingly, the most clonally diverse tumor (PS13-585) had four shared predicted neoepitopes derived from three genes. Notably, one of these was the shared fixed pathogenic *NF1*^D301N^ variant. In contrast, the majority of neoepitope candidates was present in unique or restricted numbers of sorted populations within each tumor.

**Table 1:** Predicted shared neoepitopes.

Tumor	9-Mer	Chromosome	Position	Amino acid change	Gene
PS13-9062	WARLRNPSL	chr19	48,875,815	P623S	PPP1R15A
	VFLDTISNF	chr17	81,567,467	D506N	NPLOC4
	RFSLLLFSF	chr1	78,493,345	Y201S	PTGFR
PS13-1750	QAFQVTVAI	chr1	149,934,520	M87V	MTMR11
	SAIPEGQYI	chr17	10,533,591	F741Y	MYH2
	KVRSPFETM	chr1	178,520,843	L171R	TEX35
	KTRNSFSPN	chr5	10,680,885	S146F	DAP
	YTLSLSVYI	chr7	77,922,810	R346H	PHTF2
	KLKLNPLTK	chr17	62,383,002	P8L	EFCAB3
PS13-585 Primary	KLFLNSLRK	chr17	31,200,434	D301N	NF1
	YSSVITPKI	chr11	56,375,866	M81I	OR8U1
	KILGNFLYK	chr11	56,375,866	M81I	OR8U1
	KTVSVNYIM	chr13	102,737,163	D4512N	CCDC168
PS13-585 Nodes	KLFLNSLRK	chr17	31,200,434	D301N	NF1
	YSSVITPKI	chr11	56,375,866	M81I	OR8U1
	KILGNFLYK	chr11	56,375,866	M81I	OR8U1
	KTVSVNYIM	chr13	102,737,163	D4512N	CCDC168
PS13-585 All	KLFLNSLRK	chr17	31,200,434	D301N	NF1
	YSSVITPKI	chr11	56,375,866	M81I	OR8U1
	KILGNFLYK	chr11	56,375,866	M81I	OR8U1
	KTVSVNYIM	chr13	102,737,163	D4512N	CCDC168

## Discussion

Our analysis of shared and fixed variants within the three breast cancer tumors with distinct genomic drivers and mutational landscapes highlights the extent and nature of heterogeneity within each tumor. Aneuploidy, as defined by a deviation from diploid DNA content, is a hallmark of cancer^31^. Previous flow sorting based studies have shown that cell populations with distinct DNA contents arise in premalignant stages and evolve through the natural history of neoplasias^32,33^. Furthermore, the number and distribution of ploidies within a neoplasia can vary from a single population present throughout both premalignant and invasive regions to multiple populations in a single biopsy^3,34^. We have used DNA content flow cytometry to interrogate a variety of solid tumors^30,35–38^. The genomic analyses, including NGS and CNV profiles, of sorted tumor populations enables objective identification of mutations and genomic lesions that drive the natural and clinical histories of tumors. In this current study we extended our approach to include measures of fixed and shared variants to identify both known and putative drivers of three clinically distinct breast cancers. In addition, we exploited these data to investigate patterns of predicted neoepitopes and whether genomic heterogeneity affected clinical markers. The availability of multiple mapped biopsies from each tumor provides highly favorable models for this study.

In two of the cases, we show that despite extensive and variable HRD related CNV profiles BRCA2 mutant breast cancers retain shared fixed variants targeting a discrete set of genes throughout the tumor. Notably, the majorities of fixed variants are non-coding and map to regions that are subject to copy number losses (Figs. 1, 2). This includes a tumor arising in a germ line carrier PS13-9062 and another somatic tumor PS13-1750. Of significant interest in breast and other solid tumors is the role of fixed somatic variants in BRCA and related genes in the responses to PARP inhibitors and DNA damaging agents. Notably, tumors with pathogenic germ line BRCA mutations display elevated responses to PARP inhibitors^39^. However, retention of wild type alleles in affected tumors markedly affects therapeutic responses to these targeted therapies^40^. The variation in DNA content within aneuploid tumors and the evolution of clonal populations with highly aberrant CNV patterns may provide tumor cells with multiple copies of given heterogeneous variants. Discriminating those variants, either germ line or somatic, that become fixed in a tumor can establish genotypes for BRCA and other clinically relevant genes. Notably, our approach confirmed that the pathogenic BRCA2 variants were fixed within the multiple biopsies and ploidies profiled for each BRCA^mut^ tumor by shared interstitial deletions on chromosome 13 that spanned the BRCA2 locus (Figs. 1, 2). Thus, we propose that our analysis of flow sorted biopsies provides the high-resolution detection of variants and the discrimination of those that are fixed from those that co-occur with wild type alleles necessary for the advancement of precise targeted therapies.

Despite the extent and variations in CNVs, the ploidies of the tumor populations in both BRCA mutant cases were relatively stable by our flow cytometry measures of total DNA content. Strikingly the one example of ploidy variation, the co-existing 3.2 N and 3.6 N populations in PS13-1750 biopsy A3, had the highest numbers (347 and 102) of unique variants (Fig. 2A). This dramatic “big bang”-like effect is further highlighted by the separation of these two populations from the remaining 9 sorted populations and each other in our PCA results (Fig. 3B). Notably 19 of the 99 unique variants in the 3.6 N ploidy were fixed and targeted non-coding regions of genes with well characterized roles in breast cancer and a variety of tumors including *ATR*, *CHD3*, *RPS6KA2,* and *IRF3* in PS13-1750 (Fig. 3C). The presence of these shared fixed non-coding variants suggests that regulatory elements within these genes may provide select targets in the evolution of these and possibly other BRCA related tumors.

In contrast to the *BRCA2*^mut^ tumors the 18 primary and LN biopsies from the *BRCA*^WT^ PS13-585 tumor fell into 6 distinct ploidy groups. The genomes of each population had highly aberrant but homogenous CNV profiles, characterized by *SARC* amplification and homozygous deletions in *ROBO1* and *ROBO2* (Fig. 4C, Supplemental Figs. 7, 8). The mutational landscape was defined by three shared fixed pathogenic variants, *TP53*^V172D^, *PIK3CA*^H1047R^, and *NF1*^D301N^, arising in a heterogeneous background of variants including a cluster of 144 that were restricted to a single 5.8 N population (Fig. 4A). Notably one of these, *NF1*^D301N^, is a predicted neoepitope that would provide a highly favorable target for the development of a personalized immune therapy (Table 1). In addition, the identification of a co-existing shared fixed variant, *XPO4*^E140X^, suggests a novel role for this mediator of the transport of proteins and other cargo between the nuclear and cytoplasmic compartments in breast cancer pathogenesis.

### Parallel or linear evolution?

The genomes of the two BRCA2 mutant tumors displayed elevated numbers of often heterogeneous CNVs that are typical of the HRD phenotype associated with loss of BRCA function. Strikingly, the ploidy within each tumor was relatively stable suggesting that dissemination of the aneuploid populations preceded many of the unique CNVs. In contrast, the large number and variety of somatic lesions detected in PS13-585 were highly conserved within the 6 different ploidies present within the primary tissue and adjacent nodes. The genomes have a high degree of shared aberrations including deletions, amplicons, breakpoints, and mutations. The co-occurrence of these multiple lesions in each aneuploid genome suggests that they evolved from a single progenitor present within the primary tumor and were independent of nodal dissemination and pathologic staging (pN3c). Consequently, changes in ploidy resulted in parallel evolution of both primary and lymph node clonal populations. This later evolution involved driver aberrations within the primary and selection for fixation of existing and de novo variants. One of the fixed shared variants in PS13-585 was *TP53*^V172D^. Loss of function of TP53 is associated with the appearance of aneuploid populations, as measured by DNA content flow cytometry, in esophageal neoplasias^3,41,42^. The presence of multiple aneuploid populations with highly aberrant but stable genomes throughout PS13-585 contrasts with the uniform ploidies and unstable CNV pattern of the two BRCA2^mut^ cases.

### Clonal heterogeneity and clinical markers

Our results highlight that, strikingly, tumors with aberrant genomes characterized by multiple regions of high-level copy number gains, homozygous deletions, and distinct sets of driver mutations can also maintain stable ploidies and fixed variants. Furthermore, our systematic interrogation of these tumors contradicts the heterogeneous staining observed with established IHC and FISH clinical markers for ER, PR and ERBB2 and highlights the variety of fixed and shared genomic lesions in these three distinct breast cancer cases. Notably, we propose that discriminating fixed variants will improve the identification of clinically significant CNVs and SNVs even within a single biopsy of a heterogeneous tumor.

This study was limited to three breast cancers with samples obtained at time of surgery. Future studies combining flow sorting and genomic analyses of additional breast cancers, as well as other solid tissue tumors will identify clinically relevant fixed variants and the sources of clonal evolution (e.g. ploidy, CNVs, SNVs) in diverse genetic backgrounds. Of significant interest will be applying our methods to clinical subtypes that currently lack effective targeted therapies such as triple negative breast cancer (TNBC). In addition, an outstanding question is how efficiently fixed clinically relevant variants can be identified in single biopsies, notably those arising in advanced metastatic cases and during the emergence of resistance to targeted agents.

Our findings describe highly aberrant genomes in each case and show that tumor heterogeneity can arise through variations in ploidies, allele frequencies, and copy number aberrations. Furthermore, despite the variable sources of genomic instability and heterogeneous results for established clinical markers, our rigorous interrogation of flow sorted tumor populations distinguishes fixed variants that are shared and unique in tumor populations within each tumor. We propose that this approach will enable the identification of neoepitopes that can be exploited for the development of effective vaccines to complement therapies for heterogeneous tumors.

## Materials and methods

### Clinical samples

All samples were fresh frozen at time of surgery and obtained under approval from the Mayo Clinic Institutional Review Board prior to undertaking this study (IRB protocol 08-006579). Informed consent was obtained from all patients. All breast cancers underwent central pathologic review including Nottingham histologic scoring and were evaluated by IHC for estrogen receptor (ER), progesterone receptor (PR), and by IHC +/− FISH for Her2/neu under CLIA/CAP guidelines. All research conformed to the Helsinki Declaration (https://www.wma.net/policies-post/wma-declaration-of-helsinki-ethical-principles-for-medical-research-involving-human-subjects/).

### Flow cytometry

Biopsies were minced in the presence of NST buffer and DAPI according to published protocols^3,42,43^. Prior to sorting each sample was filtered through a 35 µm mesh and collected into a 5 ml polypropylene round bottom tube. The mesh was rinsed with 750 µl of NST/10% fetal bovine serum and placed on ice while processing remaining samples. The total volume in the tube for each sample was approximately 1.5 ml. An equal volume of 20 µg/ml DAPI was added to each tube to achieve a final concentration of 10 µg/ml DAPI for flow sorting with a BD Influx cytometer with ultraviolet excitation (Becton–Dickinson, San Jose, CA, USA). The optimal settings for sorting samples with the Influx sorter were as follows: Drop formation was achieved with piezzo amplitude of 4–7 V and a drop frequency of 29.4–29.6 kHz. The sort mode was set to “one drop pure” mode with a drop delay of 31.5–32. Sheath fluid pressure was typically 17.2–17.8 psi with a 100 µm nozzle tip. For single parameter DNA content assays DAPI emission was collected at > 450 nm. DNA content and cell cycle were then analyzed using MultiCycle (Phoenix Flow Systems, San Diego, CA, USA).

For single nucleus sorting a 96 well plate sort is done using 100 µl nozzle tip and 20 psi with a frequency 29.4 kHz. Nuclei are sorted in a “one drop single” mode (high purity and high recovery of the sample) at a rate of 1500–2000 events per second to maintain purity of ≥ 98% and intactness of sorted material. Population distribution analysis and phase reproducible measurements for each sorting assay were based on 10,000–20,000 acquired events. To assure sorting single nucleus into each well of a 96 well plate both coincident particles and the position of particles inside droplets of the flow stream were monitored. Coincidence occurs when two or more particles are closer than the spacing of the droplets, so that more than one particle ends up in a single droplet.

### 96 well sort and plate alignment

The WDU (Well Deposition Unit) of the Influx is aligned to the TempPlate semi-skirt 0.2 ml, 96 well PCR plate. A test sort drop is distributed to align the stream with the center of the 96 well plate. The step is repeated several times until the test drop hits the center of the well to ensure accurate distribution of the sorted material. Then a test drop with 50 events is sorted into all wells of the 96 well plate to insure there are no skipped wells or inaccurate cell number. Sorts are done directly into a pre-filled, aligned 96 well plate with 4 µl of PBS sc. For the negative control (NC) an empty drop is sorted into the first 4 wells of the last column of the 96 well plate. Positive controls (PC) of 1000 nuclei of the population of interests are sorted into the remaining 4 wells of the last column of the 96 well plate. To avoid cross contamination during the subsequent sort the entire last column containing the NC and PC controls is covered with the adhesive sealing foil. The single nucleus is sorted into the remaining wells of the 96 well plate. After the sort is done the plate is spun down for 60 s at 900×*g*. The samples in each plate are then processed for whole genome amplification using the REPLI-g UltraFast Kit from QIAGEN according to supplier’s instructions.

### DNA extraction

DNA from sorted nuclei was extracted using an amended protocol from QIAamp^®^ DNA Micro Kit from Qiagen (Valencia, CA, USA). Briefly each sorted sample was suspended in 180 µl buffer ATL and 20 µl proteinase K (20 mg/ml) then incubated for 3 h at 56 °C for complete lysis. Samples were bound and washed according to QIAamp^®^ DNA Micro Kit instructions, eluted into 50 µl of H_2_O, then precipitated overnight with 5 µl 3 M sodium acetate and 180 µl 100% EtOH. Each sample was then centrifuged for 30 min at 20,000×*g*, washed in 1 ml of 70% EtOH for 30 min at 20,000×*g*. The samples were carefully decanted, and the DNA pellet was dried by speed vacuum then resuspended in a small volume (e.g. 10–50 µl) of H_2_0 for final concentrations suitable for accurate quantitation.

### aCGH analysis

DNAs were treated with DNAse 1 prior to Klenow-based labeling. High molecular weight reference templates were digested for 30 min while the smaller fragmented FFPE-derived DNA samples were digested for only 1 min. In each case 1 µl of 10× DNase 1 reaction buffer and 2 µl of DNase 1 dilution buffer were added to 7 µl of DNA sample and incubated at room temperature then transferred to 70 °C for 30 min to deactivate DNase 1. Sample and reference templates were then labeled with Cy-5 dUTP and Cy-3 dUTP respectively using a BioPrime labeling kit (Invitrogen, Carlsbad, CA, USA) according to our published protocols^31^. All labeling reactions were assessed using a Nanodrop assay (Nanodrop, Wilmington, DE, USA) prior to mixing and hybridization to CGH arrays (Agilent Technologies, Santa Clara, CA, USA) for 40 h in a rotating 65 °C oven. All microarray slides were scanned using an Agilent 2565C DNA scanner and the images were analyzed with Agilent Feature Extraction version 10.7 using default settings. The aCGH data was assessed with a series of QC metrics then analyzed using an aberration detection algorithm (ADM2)^44^. The latter identifies all aberrant intervals in each sample with consistently high or low log_2_ ratios based on the statistical score derived from the average normalized log_2_ ratios of all probes in the genomic interval multiplied by the square root of the number of these probes. This score represents the deviation of the average of the normalized log_2_ ratios from its expected value of zero and is proportional to the height *h* (absolute average log_2_ ratio) of the genomic interval, and to the square root of the number of probes in the interval. All aCGH data in this paper have been deposited at the National Center for Biotechnology Information Gene Expression Omnibus (GSE172262).

### Read processing and mapping

We stripped all BAM format reads from all samples using the STRIP_READS module in XYalign with the parameter “—chromosomes ALL”^45^. This module wraps uses SAMtools^46^ and repair.sh and shuffle.sh in the BBTools suite^47^ to strip, sort, and fix pairing information for reads by read group and output FASTQ files. As all samples were derived from females and the sequence similarity between the human X and Y chromosomes can affect variant calling^45^, we then used the PREPARE_REFERENCE module in XYalign to generate XX reference genomes for the hg38 human genome assemblies^48^, in which the Y chromosome was hardmasked. For all subsequent steps, we independently mapped reads, processed alignments, called variants, and conducted analyses on the modified hg38 (hg38_XX) assembly.

For each sample, we then mapped reads using BWA MEM^49^, marked duplicates using SAMBLASTER^50^, fixed pairing information with SAMtools fixmate, sorted alignment files by coordinate using SAMtools sort, and indexed sorted BAM files with SAMtools index^46^. Then, following the GATK Best Practices workflow^51,52^, we used GATK^53^ to recalibrate base quality scores and realign around indels. For both processes, we used the Mills_1000G Gold Standard Indels from the Broad Institute’s resource bundle^51^. For base quality score recalibration, we additionally included dbSNP VCF files^54^—(version 146 for hg38_XX)—also from the Broad Institute’s resource bundle^51^.

### Somatic mutation analysis

We used VarScan version 2.3.9 to call variants with the following thresholds: minimum coverage of 10, minimum variant allele frequency of 0.08, and somatic *p* value of 0.05^55^. We applied the command *somaticFilter* from VarScan to remove clusters of false positives and SNV calls near indels. We also applied the false positive filter from VarScan called *fpfilter*. Variants were annotated using variant effect predictor (VEP) version 86^56^. We used the *SNPRelate* package in R to perform the Principal Component Analysis using the *snpgdsPCA* function^57^. We used the library *upsetR* in R to generate the upset plots for visualizing the overlap in somatic mutations^58^. For the fixed somatic mutations analysis, we identified somatic mutations that are fixed by subsetting the mutations where the genotype is 1/1.

### HLA typing

We used HLA-LA for HLA typing^59^). Because HLA-LA implements a graph alignment model that requires the reads being mapped to the reference genome supported by HLA-LA. At the time of analysis, the 1000 Genome version of GRCh38 was supported. Therefore, for HLA typing purposes, we remapped the stripped reads to the 1000 Genome version of GRCh38. We then used the mapped BAM file to perform HLA typing using HLA-LA.

### Neoepitope prediction

We generated peptides consisted of 21 amino acids long using pVACseq^60^. Because Wells et al. (2020)^61^ found that neoepitopes can be predicted by using the following thresholds: binding affinity less than 34 nM, binding stability greater than 1.4 h, and tumor abundance greater than 33 TPM, we based our predictions of potential neoepitopes based on these criteria. Because we did not have whole transcriptome data, we only used the binding affinity and binding stability criteria. We computed binding affinity using artificial neural networks in netMHCpan^62^. We defined a peptide to be a potential neoepitopes if its binding affinity is less than 34 nM^61^.

## Data and code availability

We implemented the complete assembly and analysis pipeline, containing all steps described above, in Snakemake^63^ and used Bioconda^64^ to manage software. All files required to run this pipeline, including the complete conda environment (with program and package version numbers), are hosted on two Github repositories: (1) for exome processing and assembly (https://github.com/thw17/Mayo_breast_cancer_heterogeneity_assembly) and (2) for somatic variant calling and downstream analyses (https://github.com/SexChrLab/Mayo_breast_regional_heterogeneity).

## Supplementary Information

Supplementary Information.

Supplementary Legends.
