# Supplementary material for: Unique evolutionary trajectories of breast cancers with distinct genomic and spatial heterogeneity
Source: Sci Rep. 2021 May 19;11:10571. doi: 10.1038/s41598-021-90170-1 (PMC8134446; doi:10.1038/s41598-021-90170-1)
Supplement: Supplementary file 1 — Supplementary Information. [file 41598_2021_90170_MOESM1_ESM.pdf]

Unique evolutionary trajectories of breast cancers with distinct genomic and spatial heterogeneity.

Tanya N. Phung[1,2], Tim Webster[1,3], Elizabeth Lenkiewicz[4], Smriti Malasi[4], Mariacarla Andreozzi[4], Ann E. McCullough[5], Karen S. Anderson[6], Barbara A. Pockaj[7], Melissa A. Wilson[1,2*] and Michael T. Barrett[4*].

[1]School of Life Sciences, Arizona State University, Tempe, Arizona, USA

[2]Center for Evolution and Medicine, Arizona State University, Tempe, Arizona, USA

[3]Department of Anthropology, University of Utah, Salt Lake City, Utah, USA

[4]Division of Hematology/Oncology, Department of Internal Medicine, Mayo Clinic, Scottsdale, AZ USA

[5]Department of Pathology and Laboratory Medicine, Mayo Clinic in Arizona, Scottsdale, Arizona, USA

[6]Biodesign Institute, Arizona State University, Tempe, Arizona, USA

[7]Division of General Surgery, Section of Surgical Oncology, Mayo Clinic in Arizona, Phoenix, Arizona, USA

Keywords: Tumor heterogeneity, flow sorting, clonal analysis, breast cancer, BRCA2

[*] Corresponding authors:

E-mail: Barrett.michael@mayo.edu

PS13-9062: Invasive Ductal Carcinoma, Grade 3, ER+, PR+, Her2-, Node -ve

A)

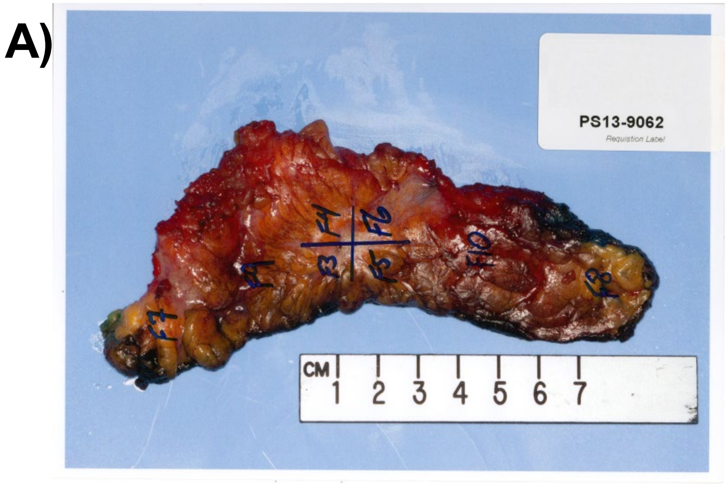

	Biopsy	Ploidy (N)
F3	Primary inferior posterior	3.7
F4	Primary inferior anterior	3.7
F5	Primary superior posterior	3.7
F6	Primary superior anterior	3.7

B)

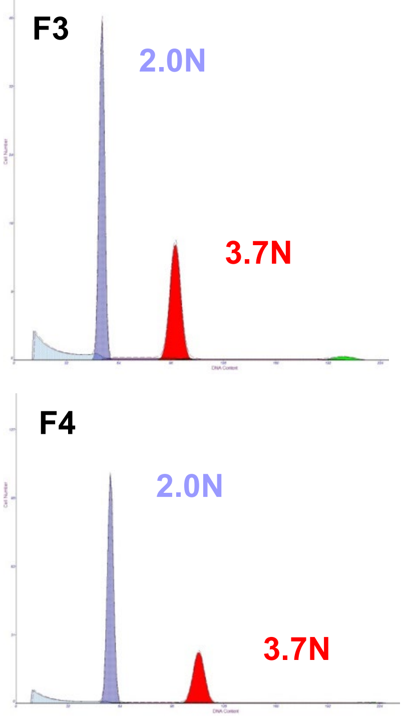

PS13-1750: Infiltrating Ductal Carcinoma, Grade 3, ER+, PR+, Her2 (3^+), Node -ve

A)

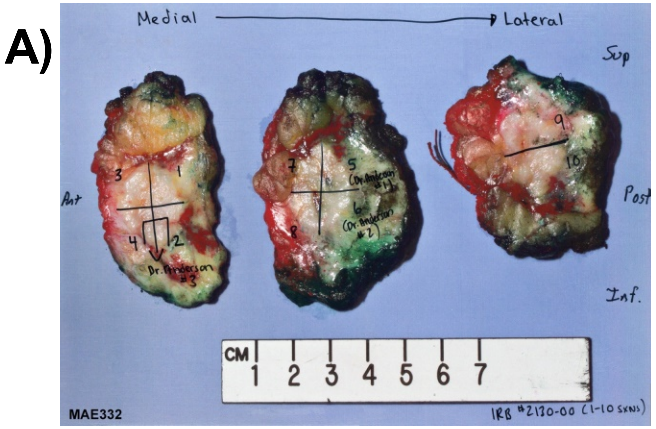

Biopsy		Ploidy(N)
A1	Mirror image	3.2
A2	Medial margin	3.2, 3.6
A3	Medial edge	3.2
A4	Medial edge	3.2
A5	Medial edge	3.2
A6	Adjacent to tumor	3.2
A7	Adjacent to tumor	3.2
A8	Mid portion	3.2
A9	Lateral aspect	3.2
A10	Lateral margin	3.2

B)

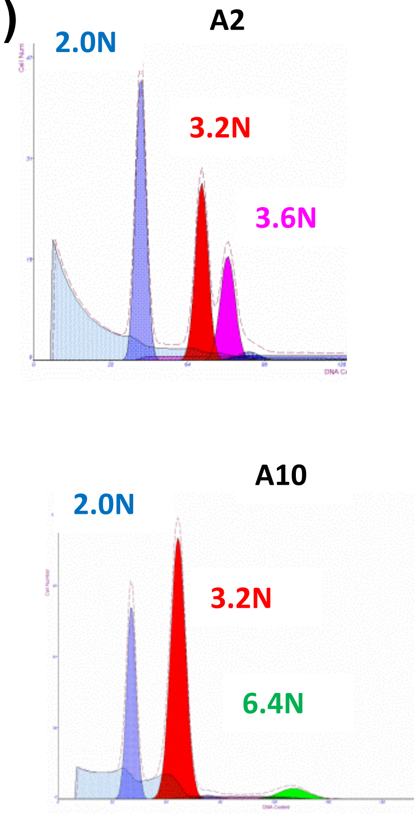

PS13-585: Infiltrating Ductal Carcinoma, Grade 3, ER+, PR-, Her2 (3+)

A)

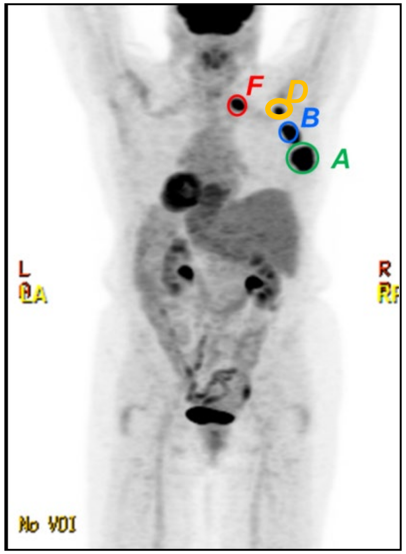

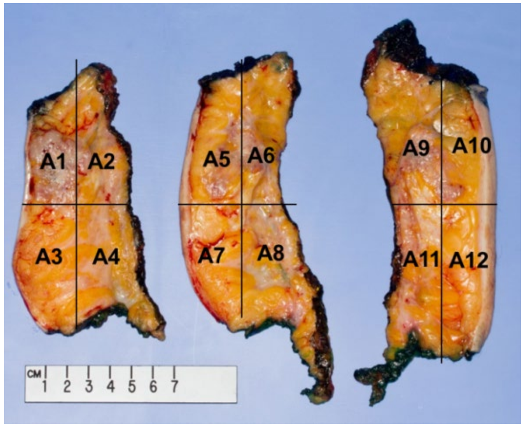

Mapping biopsies in primary tissue

B)

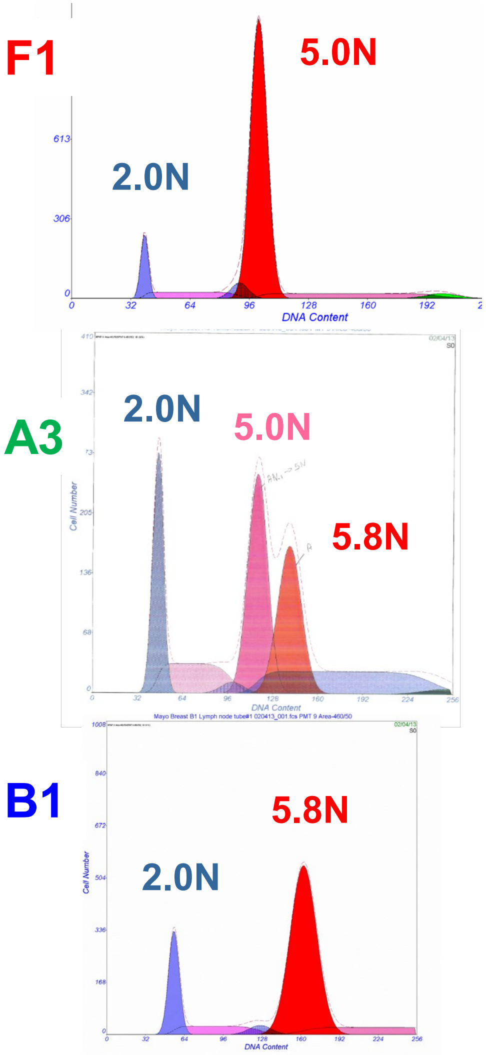

C)

Biopsy		Ploidy(N)
A1	Right Breast Tumor	5.8
A2	Right Breast Tumor	5.8
A3	Right Breast Tumor	5.8 & 5.0
A4	Right Breast Tumor	5.8 & 5.0
A5	Right Breast Tumor	5.8
A6	Right Breast Tumor	5.8
A7	Right Breast Tumor	5.8
A8	Right Breast Tumor	5.2 & 6.0
A9	Right Breast Tumor	5.8
A10	Right Breast Tumor	6.0 5.8 & 3.7
A11	Right Breast Tumor	5.8 & 3.3
A12	Right Breast Tumor	5.8 & 3.3
B1	Axillary Node	5.8
B2	Axillary Node	5.8
B3	Axillary Node	5.0
D1	Axillary Node	5.0
F1	Supraclavicular Node	5.0
F2	Supraclavicular LN F2	5.0

- 6 different ploidies
- 25 populations
- 18 biopsies
- Primary (12), adjacent nodes (4), distant node (2)
- Chemoradiation naive

Phung et al., Supplemental Figure 3

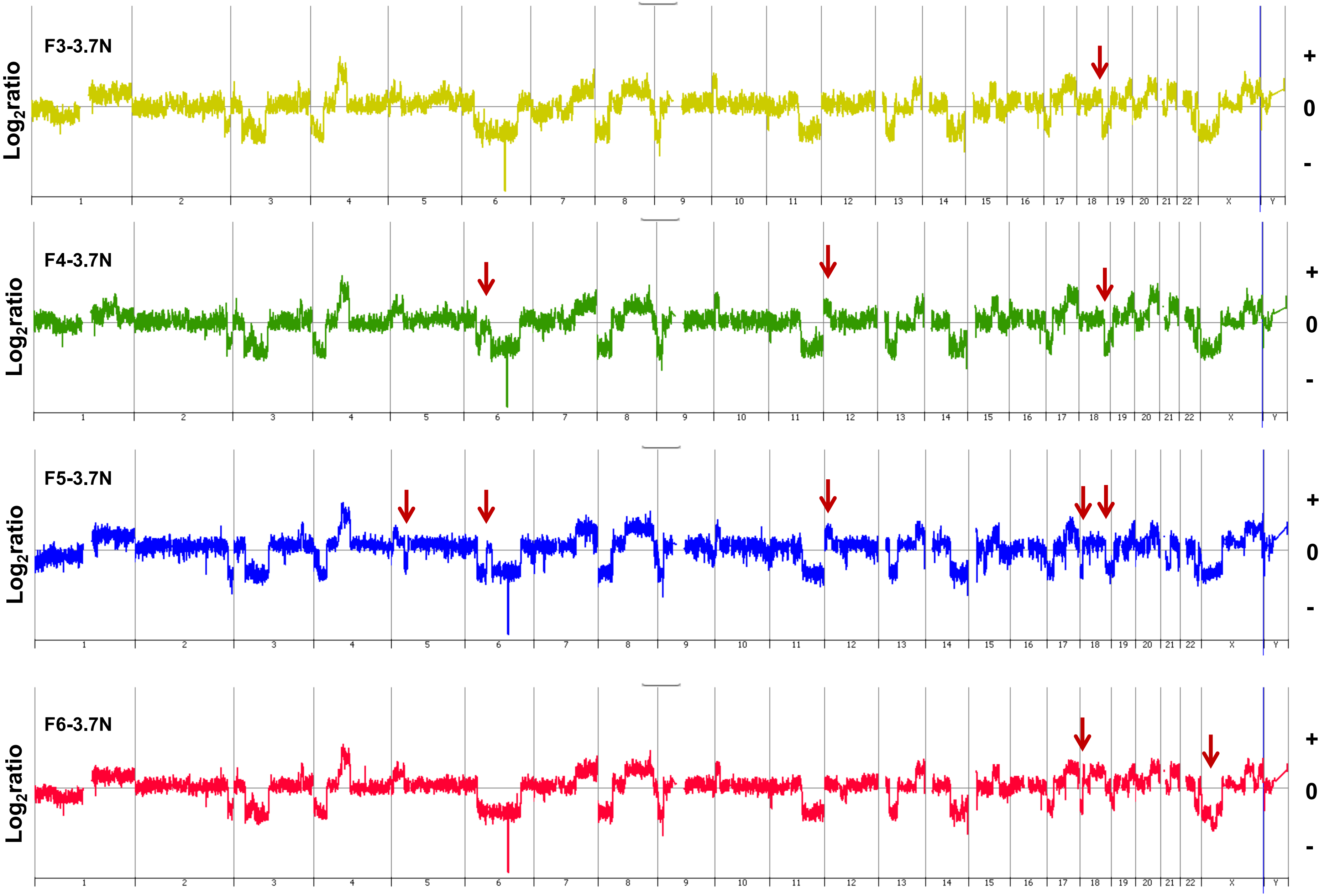

Phung et al., Supplemental Figure 4

NUMB

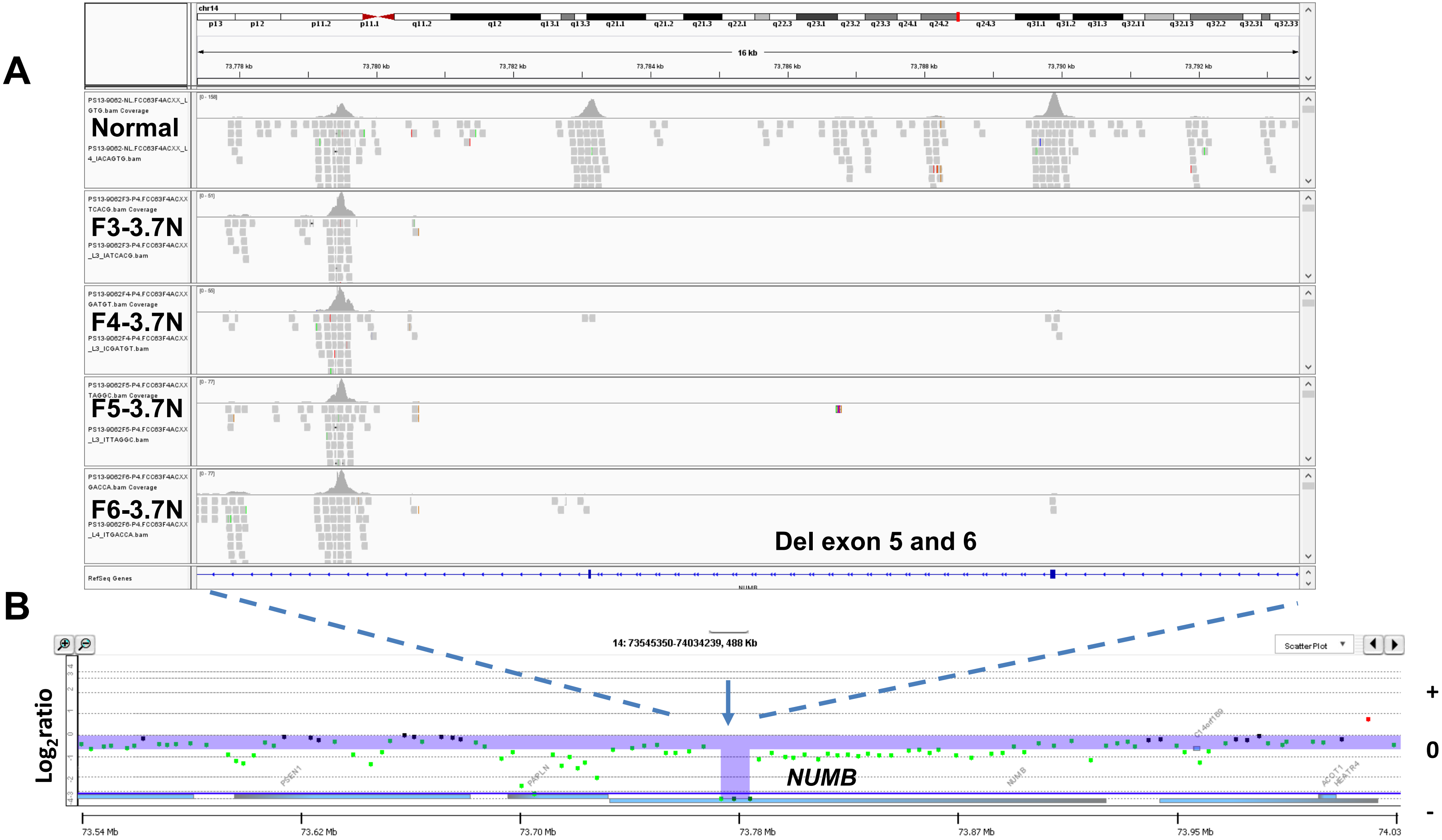
A
Normal
F3-3.7N
F4-3.7N
F5-3.7N
F6-3.7N
Del exon 5 and 6
RefSeq Genes
B
14: 73545350-74034239, 488 Kb
Log2ratio
NUMB
+
0
-
73.54 Mb
73.62 Mb
73.70 Mb
73.78 Mb
73.87 Mb
73.95 Mb
74.03

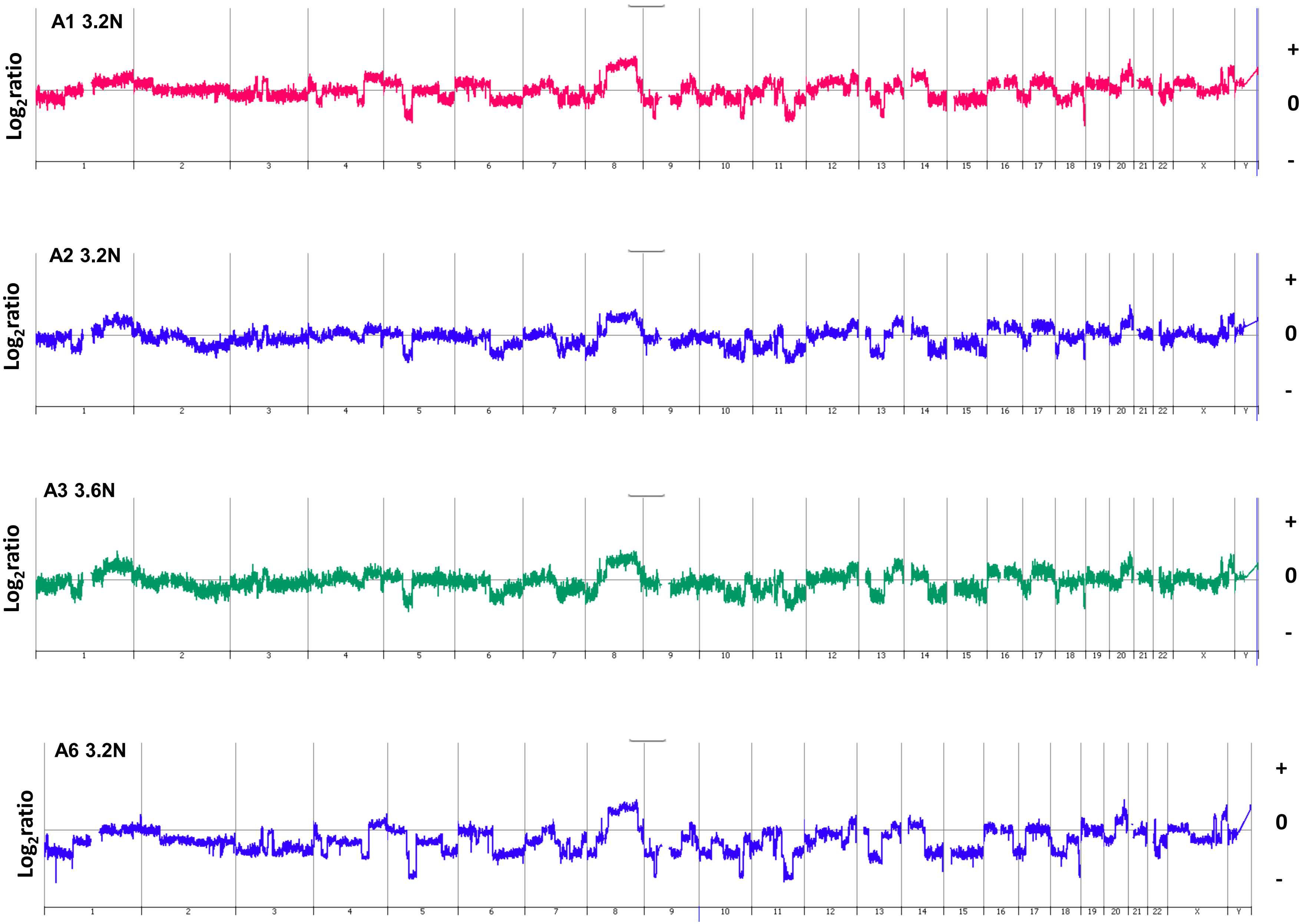

Phung et al., Supplemental Figure 6

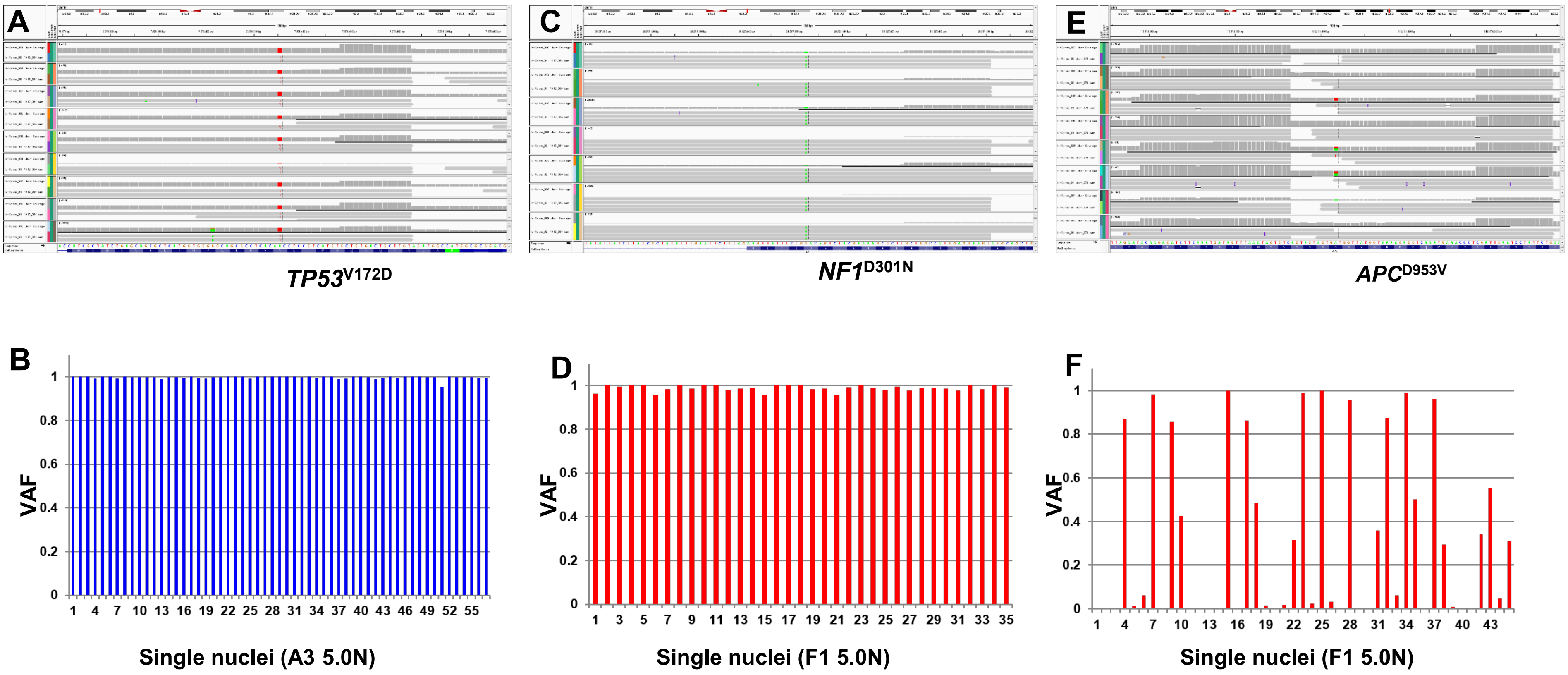

Phung et al., Supplemental Figure 7

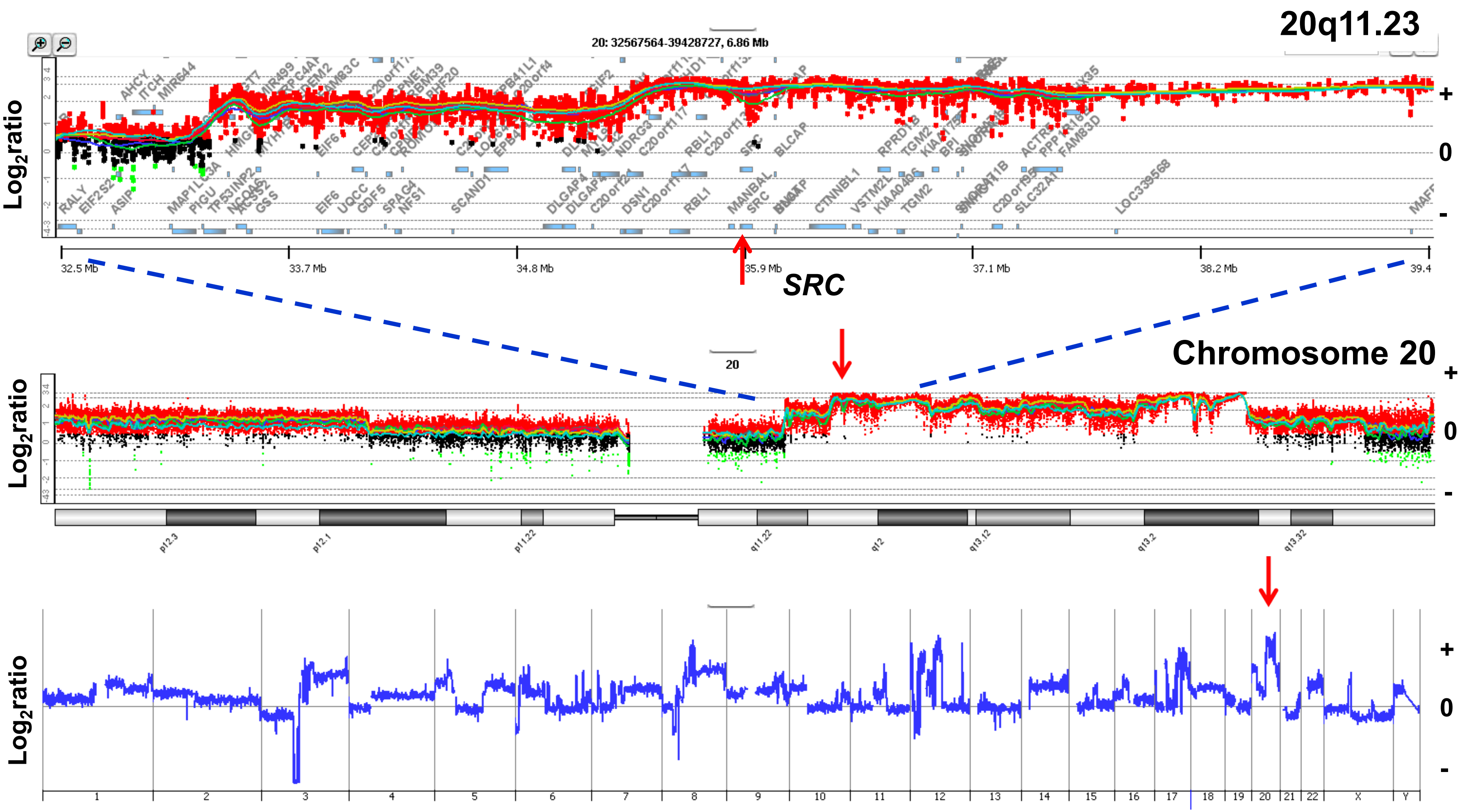

Phung et al., Supplemental Figure 8

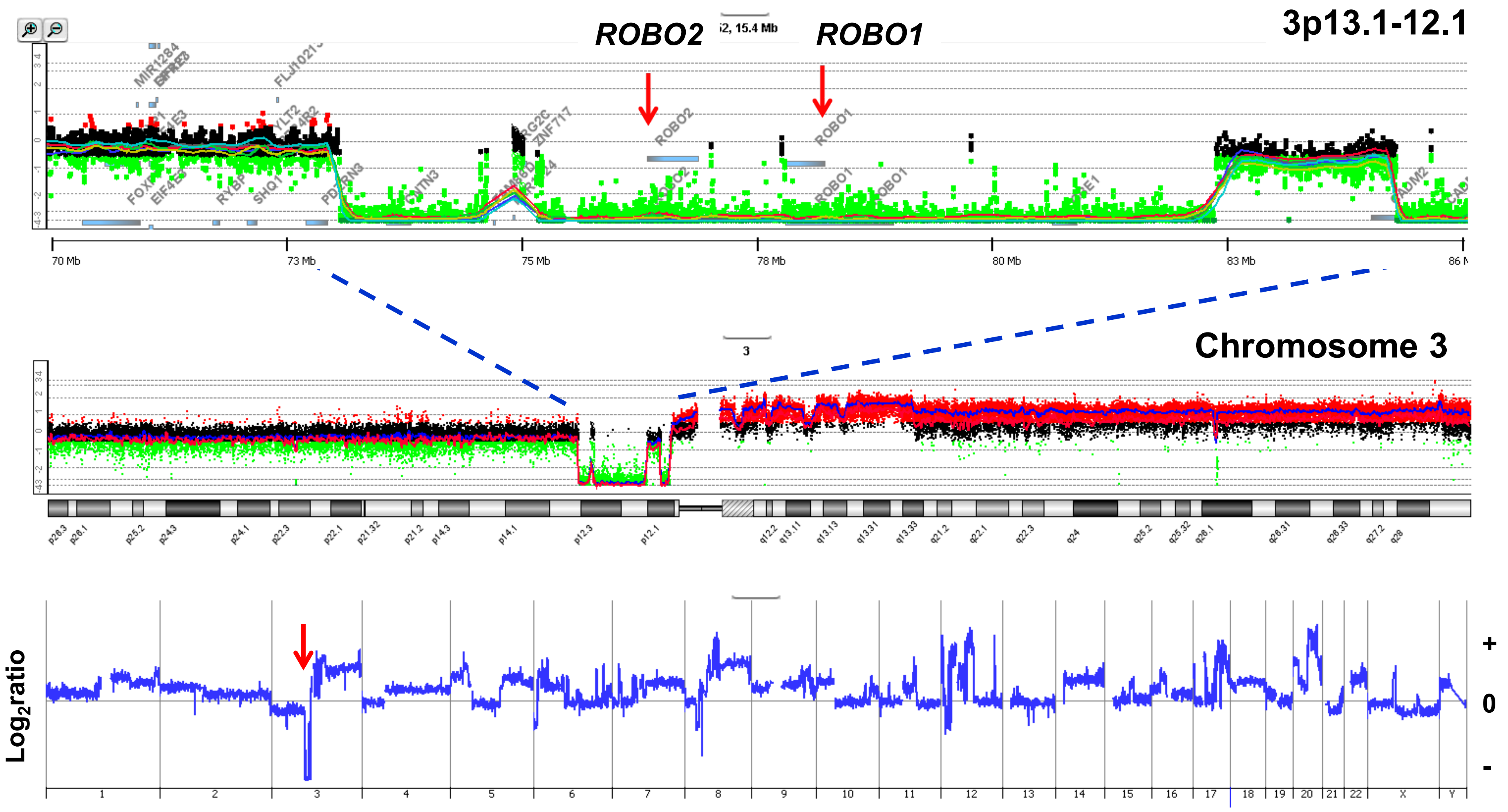

Phung et al., Supplemental Figure 9
