# Supplementary material for: Unique evolutionary trajectories of breast cancers with distinct genomic and spatial heterogeneity
Source: Sci Rep. 2021 May 19;11:10571. doi: 10.1038/s41598-021-90170-1 (PMC8134446; doi:10.1038/s41598-021-90170-1)
Supplement: Supplementary file 2 — Supplementary Legends. [file 41598_2021_90170_MOESM2_ESM.docx]

**Supplemental Figure Legends**

**Supplemental Figure 1.** **Biopsy map and flow histograms of PS13-9062**. A) Tumor tissue with co-ordinates of four mapped biopsies (F3-F6). B) DNA content flow histograms of 3.7N ploidies in biopsies F3 and F4. X-axis DNA content; y-axis number of events.

**Supplemental Figure 2. Biopsy map and flow histograms of PS13-1750**. A) Tumor tissue sections with co-ordinates of mapped biopsies (A1-A10). B) DNA content flow histograms of 3.2N and 3.6N ploidies in biopsies A2 and A10. X-axis DNA content; y-axis number of events.

**Supplemental Figure 3.** **Biopsy map and flow histograms of PS13-585**. A) Tumor tissue sections with co-ordinates of mapped biopsies in primary (A1-A12) and lymph node tissues (B, D, F). B) DNA content flow histograms of 5.0N and 5.8N ploidies in biopsies F1, A3, and B1. X-axis DNA content; y-axis number of events. C) Summary of ploidies within 18 biopsies.

**Supplemental Figure 4. CNV profiles of 3.7N populations of PS13-9062.** The genomes of each sorted populations contained unique and shared CNVs (red arrows) that reflect the *BRCA2*^mut^ genomes.

**Supplemental Figure 5. Homozygous deletion of NUMB in PS13-9062.** Fixed shared deletion of exons 5 and 6 in NUMB was detected in flow sorted 3.7N populations by both A) whole exome and B) whole genome CNV analysis. Blue shaded area denotes ADM2 step gram deletion.

**Supplemental Figure 6. CNV profiles of 3.2N and 3.6N populations of PS13-1750.** The genomes of each sorted populations contained unique and shared CNVs that reflect the *BRCA2*^mut^ genomes.

**Supplemental Figure 7. Single nucleus resequencing of TP53, NF1 and APC in flow sorted 5.0N and 5.8N populations of PS13-585.** Single nuclei from 5.0N and 5.8N populations were flow sorted into 96 well plates then resequenced for TP53, NF1, and APC using ION Torrent. A) IGV view of *TP53*^V172^ in single 5.0N nuclei sorted from A3. B) Variant allele frequency (VAF) of each single nucleus. C) IGV view of *NF1*^D301N^ in single 5.0N nuclei sorted from F1. D) Variant allele frequency (VAF) of each single nucleus. E) IGV view of *APC*^D953V^ in single 5.0N nuclei sorted from F1. F) Variant allele frequency (VAF) of each single nucleus.

**Supplemental Figure 8. CNV profiles of 5.0N and 5.8NN populations of PS13-585.** The genomes of each sorted populations contained a high-level amplicon at 20q that included the *SARC* locus 20q11.23.

**Supplemental Figure 9. CNV profiles of 5.0N and 5.8NN populations of PS13-585.** The genomes of each sorted populations contained a fixed deletion at 3p13.1-p12.1 that included the *ROBO1* and *ROBO2* loci.
